# Klippel-Trénaunay Syndrome with Intracranial Arteriovenous Malformation: A Rare Presentation

**DOI:** 10.1155/2014/202160

**Published:** 2014-02-06

**Authors:** Mahniya F. Sadiq, Waqas Shuaib, Muhammad H. Tiwana, Jamlik-Omari Johnson, Faisal Khosa

**Affiliations:** ^1^Department of Radiology and Imaging Sciences, Emory University Hospital, Atlanta, GA, USA; ^2^Emory Clinical Cardiovascular Research Institute, Atlanta, GA, USA

## Abstract

Klippel-Trénaunay syndrome (KTS) is a rare vascular congenital anomaly affecting less than 200,000 people in the United States. Vascular malformations associated with KTS tend to affect slow flow systems: venous, capillary, and lymphatic systems. The nature of the syndrome leads to a higher risk for the development of arteriovenous malformations. Our case presentation describes a patient with KTS and an associated rare presentation of intraventricular arteriovenous malformation (AVM).

## 1. Introduction

In 1900, Klippel-Trénaunay syndrome (KTS) was first described and identified by two French scientists named Maurice Klippel and Paul Trénaunay. Later, in 1907, a German-British physician named Frederick Weber identified similar cases as described by Klippel and Trénaunay [1]. For this reason, this syndrome is referred to as either Klippel-Trénaunay-Weber syndrome or KTS. This is a rare syndrome that involves a congenital malformation of veins, capillaries, and/or lymphatics, which leads to soft tissue hypertrophy and port-wine stains. These three descriptions serve to show the main features of the syndrome, of which two out of three confirm the diagnosis. The cause of KTS remains unknown, but it is believed to be the result of an intrautero insult in early gestation [2]. The severity of this syndrome presents with a wide spectrum of findings from asymptomatic cosmetic defects to debilitating hypertrophy of the limbs [3]. Patients with KTS often develop arteriovenous malformations (AVMs); however, only about 10% of these AVMs affect the head and neck [4].

In this paper, we describe a rare case where a patient with KTS is found with a massive intraventricular AVM.

## 2. Case Presentation

A 42-year-old female presented to the emergency department with trauma to the head. Upon questioning the nature of trauma, it was found that she slipped in the bathroom and hit her head on the edge of the bathtub. Physical examination of the patient was unremarkable apart from a bruise and swelling on the forehead for which the patient is seeking medical care.

**Figure 1:**
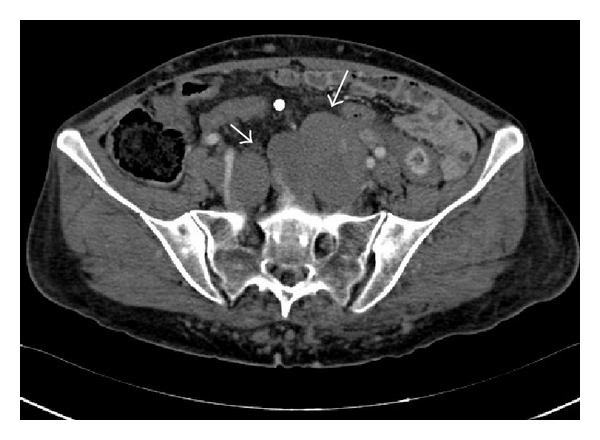
Axial contrast enhanced (arterial phase) CT of the pelvis demonstrating abnormal dilated lymphatic channels (arrows).

**Figure 2:**
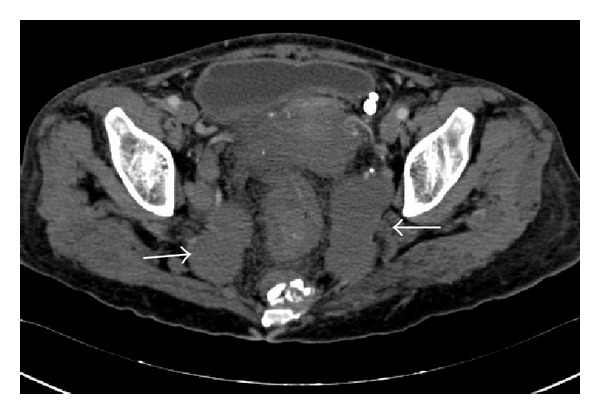
Axial contrast enhanced (arterial phase) CT of the pelvis demonstrating abnormal dilated lymphatic channels (arrows).

**Figure 3:**
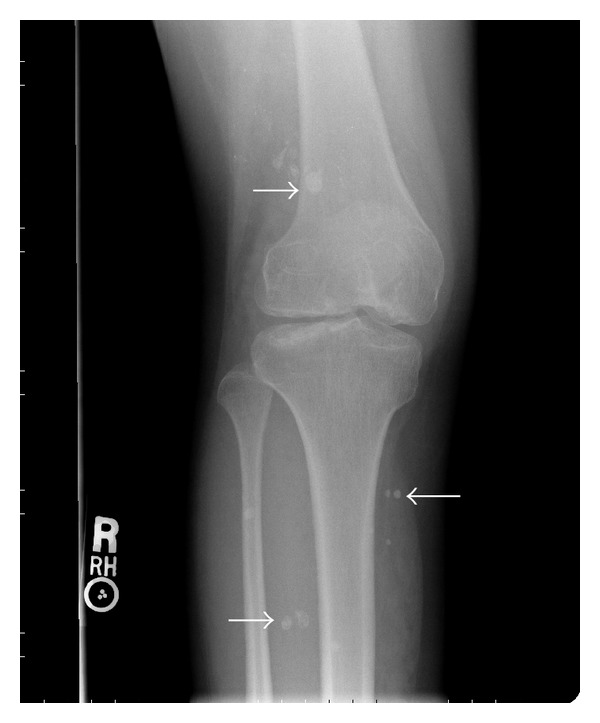
Anteroposterior radiograph of the knee demonstrating multiple venous calcifications (phleboliths) (arrows).

**Figure 4:**
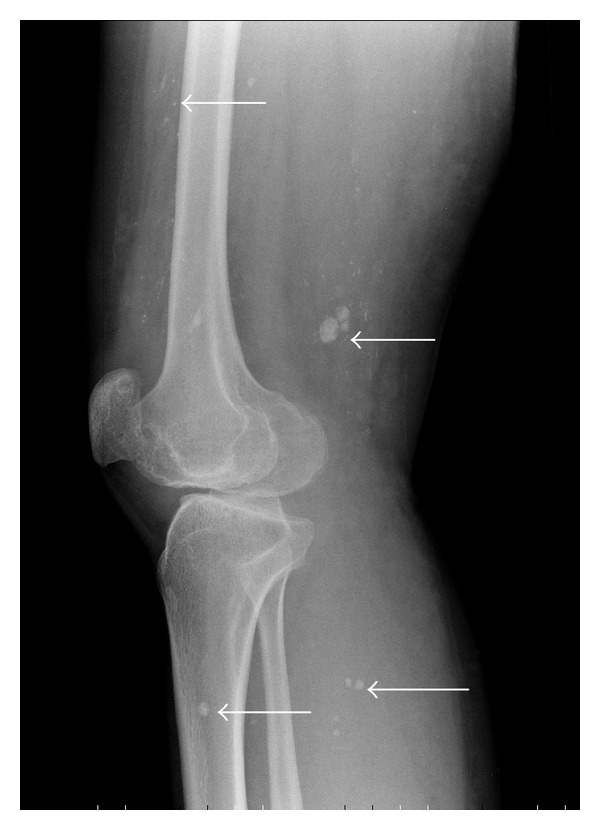
Lateral radiograph of the knee demonstrating multiple venous calcifications (phleboliths) (arrows).

Her past medical history consisted of KTS, diagnosed during childhood. On a prior visit one year ago, she was documented to have lymphatic malformations on her pelvic computed tomography (CT) (Figures 1 and 2) along with venous phleboliths on the X-rays of her right lower limb (Figures 3 and 4).

**Figure 5:**
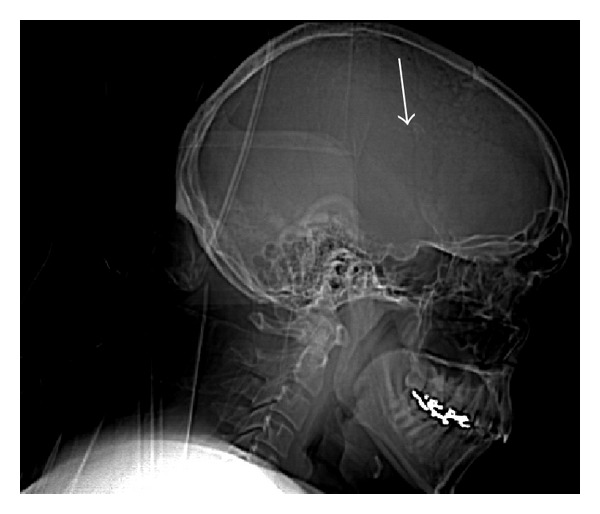
Lateral radiograph of the skull demonstrating a focus of calcification in the frontoparietal region (arrow).

**Figure 6:**
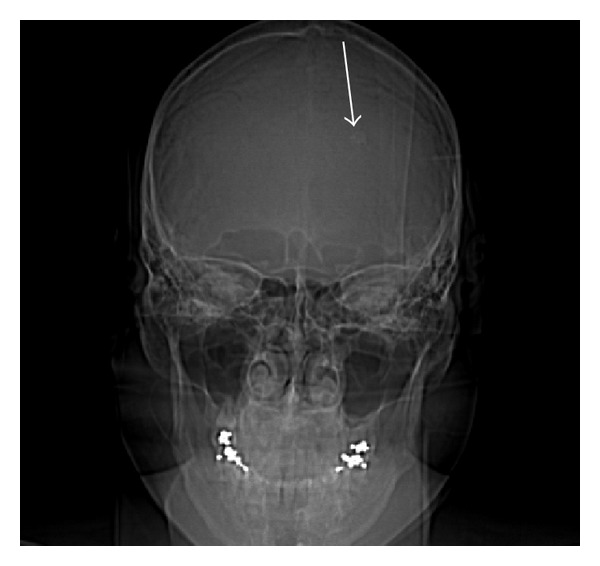
Anteroposterior radiograph of the skull demonstrating a focus of calcification to the left of the midline (arrow).

**Figure 7:**
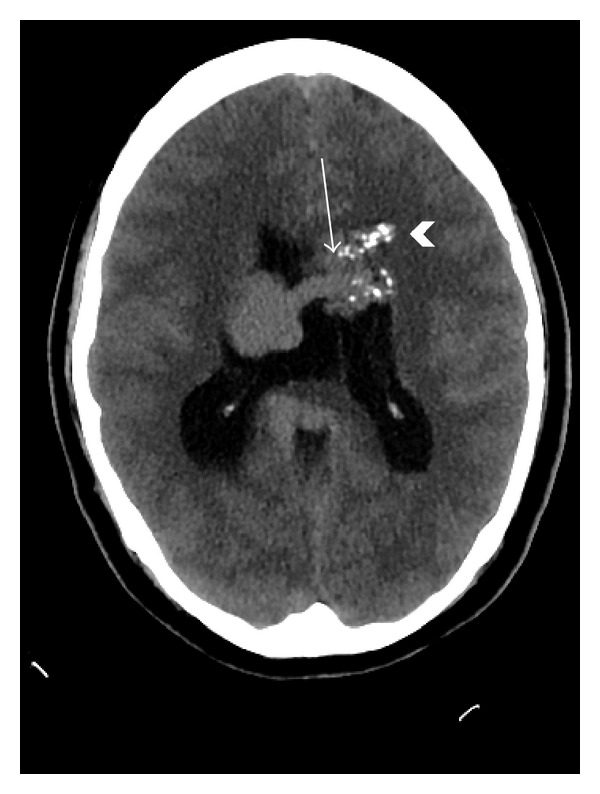
Axial noncontrast enhanced CT of the brain demonstrating a serpiginous intraventricular structure (arrow) with calcifications (arrowhead).

**Figure 8:**
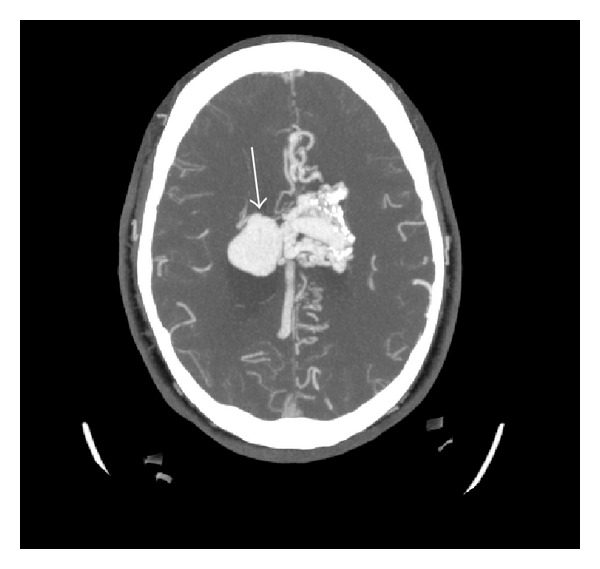
Axial contrast enhanced CTA (MIP) of the brain demonstrating a serpiginous intraventricular enhancing structure (arrow).

**Figure 9:**
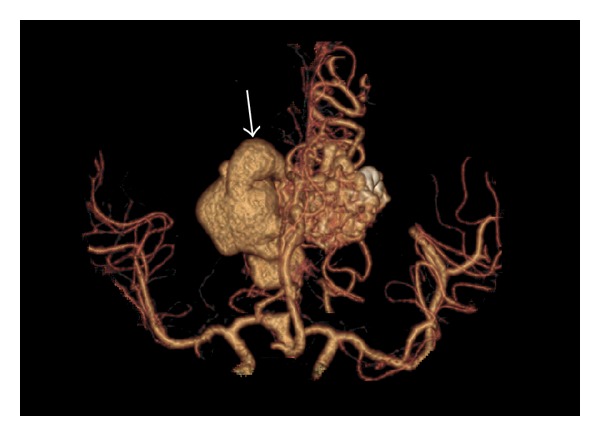
Axial contrast enhanced CTA (MIP) surface shaded image of the brain demonstrating a serpiginous intraventricular enhancing structure (arrow). The AVM is supplied by branches of the anterior cerebral artery and drains via superior sagittal sinus.

As part of the workup for the bruise on her head, a two-view X-ray of the head was conducted which showed calcifications in the frontoparietal region and to the left of the midline (Figures 5 and 6). As a result of the radiographic findings, a CT of the head (with and without contrast) was acquired which showed an intraventricular AVM (Figures 7 and 8). Maximum intensity projection on a CT angiography was done which further clarified the nature of the intracranial lesion (Figure 9).

The diameter of the AVM was found to be over 6.4 cm. In view of this information, the cerebral AVM was classified as grade 4 according to the grading scale described by Ogilvy et al. (Table 1) [5]. Due to the location and high grade of the AVM, the patient was referred to neurosurgery as a precautionary therapeutic measure.

## 3. Discussion

Arteriovenous malformations (AVMs) are commonly seen in patients with Klippel-Trénaunay syndrome (KTS); however, its subcategory of intracranial AVMs is very rare with less than 1% of the general population being affected [5–7]. To date, there are a limited number of reported cases of KTS and associated intracranial AVMs [2, 4]. While more than 50% of intracranial AVMs result in an intracranial hemorrhage, it may also include symptoms such as seizures, headaches, and progressive numbness [8, 9].

A previous study correlates middle age with stabilization of intracranial AVMs so they remain asymptomatic and are therefore not treated [7]. A similar scenario is seen in the aforementioned patient who was asymptomatic and presented to the ED with trauma.

After the initial diagnosis of an intracranial AVM, possible treatment plans may include embolization, radiosurgery, and craniotomy. The question of surgical intervention can be answered using the Spetzler-Martin grading system. Intracranial AVMs graded I, II, and III show favorable surgical outcomes with a 96%, 95%, and 88% chance of survival, respectively. For grades IV and V, the survival rate drops significantly down to 73% and 57%, respectively; craniotomy is therefore not recommended [4, 10–13]. Grades IV and V require a multidisciplinary approach with case-by-case analysis [5]. Less severe cases of intracranial AVMs are managed using a combined intervention of radiosurgery and embolization.

The wide spectrum of anomalies seen in KTS warrants an individualized analysis of the best treatment plan. KTS commonly includes complications such as pulmonary embolism, AVMs, thrombosis, and hyper coagulation. Therefore, it is important for physicians to be familiar with the variety of radiologic findings associated with KTS and the increased frequency of its related complications [14, 15].

## 4. Conclusion

Arteriovenous malformations (AVMs) of the head and neck are rare in Klippel-Trénaunay syndrome (KTS). In the acute setting, patients often present with seizures, headaches, and progressive numbness. While the clinical history may aid in making the diagnosis, radiologic imaging provides valuable information regarding the site, number, location, and the size of the malformation, as well as any complicating factors such as perforation. Early diagnosis has significant implications for disease management particularly surgical planning.

## Figures and Tables

**Table 1:** Spetzler-Martin AVM Grading Scale. The score is calculated by summing the points for each category.

AVM size	Adjacent eloquent cortex	Draining veins
Under 3 cm = 1	Noneloquent = 0	Superficial only = 0
3–6 cm = 2	Eloquent = 1	Deep veins = 1
Over 6 cm = 3		

## Disclaimer

There was no commercial funding for the study. The authors have full control over all the data. The paper will not be published elsewhere in any language without the consent of the copyright owners.
